# Reduced Mortality with Use of Point of Care Cell Suspension Autograft

**DOI:** 10.1093/jbcr/iraf221

**Published:** 2025-12-01

**Authors:** Muzamil Ahmad, Soman Sen, Kathleen Romanowski, Tina Palmieri, David G Greenhalgh, Jason Heard

**Affiliations:** Oakland University William Beaumont School of Medicine, Rochester, MI 48309, United States; Department of Surgery, University of California, Davis, Regional Burn Center, Davis, CA 95817, United States; Shriners Hospital for Children Northern California, Sacramento, CA 95817, United States; Department of Surgery, University of California, Davis, Regional Burn Center, Davis, CA 95817, United States; Shriners Hospital for Children Northern California, Sacramento, CA 95817, United States; Department of Surgery, University of California, Davis, Regional Burn Center, Davis, CA 95817, United States; Shriners Hospital for Children Northern California, Sacramento, CA 95817, United States; Department of Surgery, University of California, Davis, Regional Burn Center, Davis, CA 95817, United States; Shriners Hospital for Children Northern California, Sacramento, CA 95817, United States; Department of Surgery, University of California, Davis, Regional Burn Center, Davis, CA 95817, United States; Shriners Hospital for Children Northern California, Sacramento, CA 95817, United States

**Keywords:** cell suspension autograft, burns, mortality, treatment outcome, skin transplantation

## Abstract

Cell suspension autograft (CSA) is a non-cultured, autologous cellular suspension used in partial-thickness burns or as an adjunct to widely meshed split-thickness skin grafts (STSGs). While CSA has been shown to improve patient outcomes in burn care, literature is limited in highlighting its impact on mortality when used in combination with STSG. This retrospective, matched, case–control study investigates the clinical efficacy of CSA in adult patients with burn injuries admitted to a regional burn center from 2015 to 2023. Patients treated with CSA and STSG (*n* = 63, “CSA-treated”) were compared against patients treated with STSG alone (*n* = 126, “non-CSA-treated”). Non-CSA-treated patients were matched in a 2:1 fashion to CSA-treated patients based on third-degree burned TBSA and age. Outcomes included mortality, length of stay (LOS), intensive care unit LOS (ICU LOS), and number of procedures. Multivariate analyses revealed that CSA-treated patients had a significant reduction in mortality (*P* = .0445) and a 78.9% reduction in odds of death (OR: 0.211) compared to non-CSA-treated patients. Cell suspension autograft-treated patients displayed nonsignificant increases in LOS (*P* = .0670), ICU LOS (*P* = .0851), and number of procedures (*P* = .9084). Selection and chronology bias may partially account for the improved mortality in the CSA-treated group. The nonsignificant increases in LOS, ICU LOS, and number of procedures may be reflective of increased survivorship. These findings demonstrate that CSA enhances survival in patients with burn injuries when used with STSG, warranting further research to confirm these results.

## INTRODUCTION

Burns remain a common type of injury worldwide, with the World Health Organization (WHO) estimating 11 million burn injuries each year.^1^ Despite modern advancements in burn treatment, sepsis resulting in multiorgan failure remains the leading cause of mortality among patients with burn.^2^ Thus, burn treatment prioritizes prompt restoration of the skin through early excision and grafting.^3^ The use of autologous split-thickness skin grafts (STSGs) remains the standard of care for the closure of burn wounds.^3^ However, the use of STSG can become challenging in extensive burn injuries where viable donor skin is limited. In addition, surgically created donor sites carry a risk of infection, which can impair and delay the restoration of the skin barrier.^4^ With these considerations in mind, burn treatment balances between obtaining sufficient skin for grafting while minimizing the creation of donor sites.

The use of a cell suspension autograft (CSA) functions as an adjunct in full-thickness burns or as an alternative to the traditional autologous STSG in burn treatment. Cell suspension autograft is created intraoperatively from a small, non-cultured, split-thickness skin sample and are applied onto the wound bed using a spray-on technique.^5^ Cell suspension autograft can be used alone on partial-thickness burns or applied over a STSG with wide-meshed ratios (3:1 or 4:1) to fill interstices at a faster rate compared to STSG alone.^6^ For these reasons, CSA appears as a promising treatment in patients with more extensive burn injuries to judiciously use available donor skin and achieve higher coverage rates per operation.

While existing literature has demonstrated that CSA can reduce donor site sizes,^5^ shorten hospital stays,^7-9^ and decrease the number of surgical procedures,^7^ there remains a gap regarding its impact on patient outcomes when used in combination with widely meshed STSG, including: length of stay (LOS); number of procedures; and mortality. Therefore, the aim of this study is to evaluate the combined efficacy of CSA and widely meshed STSG after full-thickness burn excision on patient mortality, LOS, and number of surgical procedures compared to widely meshed STSG alone.

## METHODS

A registry based, retrospective, matched case control analysis was conducted on all adult patients with burn injuries admitted to a regional burn center between July 2015 and December 2023. As a retrospective analysis, this study was reviewed by the IRB staff and determined to be exempt from IRB review.

Using a de-identified burn registry, patients were divided into 2 groups: STSG + CSA (experimental group; “CSA-treated”) and STSG without CSA (control group; “non-CSA-treated”). Data points extracted from the registry included: total body surface area (TBSA) burned; second-degree TBSA; third-degree TBSA; presence of an inhalation injury; number of operations; LOS; intensive care unit length of stay (ICU LOS); and mortality. Intensive care unit-free days to day 28 were used in analysis as well instead of raw ICU LOS to reduce survivorship bias and better capture the combined effects of mortality and ICU duration. Intensive care unit-free days to day 28 (ICUFD28) was calculated for each subject and defined as the number of days a patient was alive and not in the ICU between admission and day 28. Patients who died before day 28 were assigned a value of zero ICU-free days. The primary exposure was treatment with CSA. The primary outcome was in-hospital mortality. Secondary outcomes included hospital LOS and ICU LOS.

Cell suspension autograft-treated patients were matched in a 1:2 format with non-CSA-treated patients based first on third-degree TBSA (±2% TBSA) and second on age (±2 years). A 1:2 matching ratio was utilized to increase statistical power while minimizing heterogeneity of controls, given the limited availability of comparable patients of similar age and TBSA burned. Matching was performed on third-degree TBSA rather than total TBSA because third-degree burns more accurately reflect the burden of surgically managed burn injury which was central to the study’s focus on operative techniques. Matching variables (age and third-degree TBSA) were considered a priori confounders of the relationship between CSA use and mortality. Inhalation injury was evaluated as a potential confounder but not used for matching. Statistical analysis was performed with SAS Software (Version 9.4). Univariate analysis of the matched patients was completed using the chi-squared test. Fisher’s exact test was employed when cell numbers were small. Multiple linear regression was used to analyze continuous outcomes, while multiple logistic regression was used to analyze binary outcomes. Regression models were chosen based on the lowest Akaike information criterion (AIC).

## RESULTS

In total, 189 patients were included in this study: 63 CSA-treated patients and 126 non-CSA-treated patients. Of the CSA-treated group and the non-CSA-treated group, the mean age was 48.2 and 47.6; mean total TBSA was 37.4% and 37.6%; mean second-degree TBSA was 6.2% and 6.3%; and mean third-degree TBSA was 31.2% and 31.3%, respectively (Table 1). An inhalation injury was present in 36.5% (23/63) of the CSA-treated group and 37.3% (47/126) of the non-CSA-treated group (Table 1).

**Table 1:** Patient Characteristics

**Variable**	**CSA-treated (*n* = 63)**	**Non-CSA-treated (*n* = 126)**	**Total (*n* = 189)**
Age	48.2 ± 18.0	47.6 ± 16.9	47.8 ± 17.2
Total TBSA	37.4 ± 35	37.6 ± 23.6	37.5 ± 23.7
Second-degree TBSA	6.2 ± 9.6	6.3 ± 8.6	6.3 ± 8.9
Third-degree TBSA	31.2 ± 23.3	31.3 ± 23.6	31.3 ± 23.5
Inhalation injury	23 (36.5%)	47 (37.3%)	70 (37%)
Length of stay	72.7 ± 50.6	53.7 ± 48.7	60.1 ± 50.0
ICU length of stay	62.5 ± 48.0	45.7 ± 48.0	51.3 ± 48.5
ICU free days to day 28	5.6 ± 9.5	3.2 ± 6.6	4.8 ± 8.7
Number of procedures	3.8 ± 2.9	3.5 ± 3.4	3.6 ± 3.3
Mortality	3 (4.8%)	29 (23.2%)^*^	32 (17%)^**^

Data presented as mean ± standard deviation for continuous variables and number (percentage) for binary variables. ^*^ = Out of 125 patients due to missing mortality outcome for one non-CSA-treated patient. ^**^ = Out of 188 total patients due to missing mortality outcome for one non-CSA-treated patient.

### Mortality

The mortality rate was significantly lower in the CSA-treated group at 4.8% (3/63) compared to 23.2% (29/125) in the non-CSA-treated group. One patient in the non-CSA-treated group was missing a mortality variable and was excluded. This difference was statistically significant across the chi-square test (*χ*^2^ = 10.08, *P* = .0015) and Fisher’s exact test (*P* = .0009), given the small number of deaths in the CSA group. Multiple logistic regression analysis for mortality (Table 2) revealed that the odds of death were 78.9% less for CSA-treated patients compared to non-CSA-treated patients (OR = 0.211; 95% CI, 0.046-0.962; *P* = .0445), when controlled for total TBSA, age, and LOS. In addition, multiple logistic regression revealed that increases in total TBSA burned (OR = 1.119; 95% CI, 1.073-1.167; *P* < .0001), age (OR = 1.072; 95% CI, 1.028-1.118; *P* = .0013), and LOS (OR = 0.962; 95% CI, 0.942-0.982; *P* = .0002), were significant predictors for increased mortality.

**Table 2:** Multiple Logistic Regression for Mortality (AIC of 105.4)

**Variable**	**Odds ratio**	**95% Confidence interval**	***P*-value**
Cell suspension autograft	0.211	0.046-0.962	.0445
Total TBSA	1.119	1.073-1.167	<.001
Length of stay	0.962	0.942-0.982	.0002
Age	1.072	1.028-1.118	.0013

### Length of stay

The average LOS between the CSA-treated group and the non-CSA-treated group was 72.7 days and 53.7 days, respectively (Table 1). Multiple linear regression for LOS (Table 3) revealed that total TBSA, age, and number of procedures significantly increased LOS whereas mortality significantly decreased LOS. For every 1% increase in TBSA, LOS increased by 0.8 days. For every increase in age by 1 year, LOS increased by 0.3 days. For each additional procedure a patient had, LOS increased by 9.2 days. Mortality was associated with 37.7-day reduction in LOS. Although there was no statistically significant difference in LOS between CSA-treated patients and non-CSA-treated patients, multiple linear regression showed that CSA treatment increased LOS by 8.43 days (*P* = .0670). Multiple linear regression analysis ran only with survivors found no significant difference, but the parameter estimate decreased from 8.43 days to 6.39 days (*P* = .1439).

**Table 3:** Multiple Linear Regression for Length of Stay

**Variable**	**Parameter estimate**	**Standard error**	***P*-value**
Cell suspension autograft	8.43	4.58	.0670
Total TBSA	0.766	0.13	<.001
Age	0.330	0.14	<.001
Mortality	−37.70	6.44	<.001
Inhalation injury	−5.73	5.08	.2607
Number of procedures	9.22	0.83	<0.001

Adjusted R-squared of 0.6738 with intercept of −12.0, patient with missing mortality variable excluded.

### ICU length of stay

The average ICU LOS between the CSA-treated group and the non-CSA-treated group was 62.5 days and 45.7 days, respectively (Table 1). Multiple linear regression for ICU LOS (Table 4) revealed that total TBSA, age, and number of procedures significantly increased ICU LOS whereas mortality significantly decreased ICU LOS. For every 1% increase in total TBSA, ICU LOS increased by 0.9 days. For every 1-year increase in age, ICU LOS increased by 0.4 days. For each additional procedure performed, ICU LOS increased by 8.1 days. Mortality was associated with 31.8-day reduction in ICU LOS. Although there was no statistically significant difference in ICU LOS between CSA-treated patients and non-CSA-treated patients, multiple linear regression demonstrated that CSA treatment increased ICU LOS by 7.8 days (*P* = .0851). Multiple linear regression analysis ran only with survivors showed no significant difference in ICU LOS between both groups, but decreased parameter estimates from 7.8 days to 5.0 days (*P* = .3291).

**Table 4:** Multiple Linear Regression for Intensive Care Unit Length of Stay

**Variable**	**Parameter estimate**	**Standard error**	***P*-value**
Cell suspension autograft	7.82	4.52	.0851
Total TBSA	0.87	0.13	<.001
Age	0.35	0.14	.0108
Mortality	−31.76	6.35	<.001
Inhalation injury	−2.92	5.02	.5606
Number of procedures	8.13	0.82	<.001

Adjusted R-squared of 0.6623 with intercept of −23.44, patient with missing mortality variable excluded.

### ICU-free days to day 28

The average ICU-free days to day 28 (ICUFD28) between the CSA-treated group and the non-CSA treated group was 5.6 days vs 3.2 days, respectively (Table 1). Multiple linear regression for ICUFD28 (Table 5) showed that CSA use, larger burn size and older age were significantly associated with fewer ICU free days. Cell suspension autograft treatment was associated with 2.5 fewer days of ICUFD28. For every 1% increase in TBSA ICUFD28 decreased by 0.24 days. For every year increase in age, the ICUFD28 decreased by 0.09 days.

**Table 5:** Multiple Linear Regression for ICU-Free Days up to Day 28

**Variable**	**Parameter estimate**	**Standard error**	***P*-value**
Cell suspension autograft	−2.53	1.05	.0172
Total TBSA	−0.24	0.03	<.001
Age	−0.09	0.03	.0033
Mortality	−1.04	−0.73	.4689
Inhalation injury	−1.45	1.16	.2132

Adjusted R-squared of 0.4288 with intercept of 19.73, patient with missing mortality variable excluded.

### Number of procedures

Cell suspension autograft-treated patients had an average of 3.8 procedures, while non-CSA-treated patients had an average of 3.5 procedures (Table 1). Multiple linear regression for number of procedures (Table 6) revealed that total TBSA was significantly associated with more procedures whereas mortality was significantly associated with less procedures. For every 1% increase in total TBSA, the number of procedures increased by 0.08. Mortality was associated with 1.9 less procedures. Cell suspension autograft treatment did not have a significant impact on the number of procedures (*P* = .9084). Age and inhalation injury did not have a significant impact on the number of procedures (*P* = .5854 and *P* = .0775, respectively). Multiple linear regression analysis ran only with survivors found no significant difference in the number of procedures between both groups.

**Table 6:** Multiple Linear Regression for Number of Procedures

**Variable**	**Parameter estimate**	**Standard error**	***P*-value**
Cell suspension autograft	0.05	0.41	.9084
Total TBSA	0.08	0.01	<.001
Age	−0.01	0.01	.5854
Mortality	−1.90	0.56	.0008
Inhalation injury	0.80	0.45	.0775

Adjusted R-squared of 0.3885 with intercept of 0.89, patient with missing mortality variable excluded.

## DISCUSSION

As burn care continues to evolve with the introduction of new operative approaches and critical care management, it is vital to investigate all facets of treatment to understand the full impact an approach may have on patient outcomes. The emergence of CSA into the arsenal of the burn physician has shown to be an effective tool in achieving faster healing times and reducing patient burden, all while requiring less donor skin when compared to STSG alone.^10^ In decreasing the donor skin requirement to cover larger burns, donor site associated complications can be reduced.^11^ In addition, when CSA is applied in combination with widely meshed STSG ratios, an enhanced rate of re-epithelialization is observed.^6^ Due to these positive findings, CSA application poses as a promising addition to burn treatment and to improve patient outcomes. However, literature is rather limited in demonstrating its combinatory impact on patient outcomes when used with widely meshed STSG, especially regarding mortality. The results of the current study demonstrate a surprisingly strong impact on mortality with no significant differences in the other evaluated outcomes which should be interpreted with caution.

Between CSA-treated patients and non-CSA-treated patients, mortality rates varied significantly at 4.7% and 23.2%, respectively. Notably, the CSA-treated group exhibited a 78.9% reduction in odds of death compared to the non-CSA-treated group, even after adjusting for TBSA burned, age, and LOS. Age and TBSA burned are well-established predictors of mortality in patients with burn injuries.^12^ However, because cases and controls were explicitly matched on third-degree TBSA and age, the variability, and thus predictive strength, of these variables is substantially reduced in the regression model. As a result, CSA emerges as a stronger predictor of mortality, which may partly reflect residual confounding. Inhalation injury, another known mortality predictor, was excluded from the final model based on model fit criteria (AIC), but its exclusion may further limit the model’s ability to fully account for differences in baseline risk. The observed mortality benefit observed in this retrospective study is likely multifactorial and should be interpreted with caution given the study design.

The most optimistic hypothesis is that patients achieve quicker wound closure rates and therefore have lower risk of infections, decreased metabolic demands, and less risk of complications such as death with CSA. However, there is an obvious selection bias and chronology bias that cannot be overcome by multivariate logistic modeling. Patients that are doing clinically well are more likely to undergo donor site harvesting and skin grafting whereas those not doing well will either have surgery delayed or temporizing wound coverage until they clinically improve or succumb. In addition, to properly match with controls, the database was queried to 2015 where overall care may have been inferior to the more recent years where CSA was more likely to be used, a type of chronology bias. Furthermore, the introduction and use of biodegradable temporizing matrix (BTM), notably around the same time as CSA, suggests that patients treated in the more recent years with CSA may have also benefited from BTM; however, this is not possible to discern from the registry data. Regardless of the bias described within, the reduction in mortality is still noteworthy and warrants further investigation with future studies.

In terms of LOS and ICU LOS, the CSA-treated group exhibited longer LOS and ICU LOS compared to the non-CSA-treated group, though this difference was not statistically significant. These findings starkly contrast existing studies which demonstrate a reduction in LOS for surviving patients with small and large burns treated with CSA^7-10^^,^^13^; however, these studies excluded patients that died which may explain the differences found in the current study that could not be completely accounted for by controlling for mortality in regression modeling. To address this, we incorporated ICU-free days to day 28 as a composite outcome reflecting both mortality and ICU duration. Using this more robust metric, CSA-treated patients had significantly fewer ICU-free days, suggesting prolonged ICU utilization and/or early mortality in this group. Importantly, this measure better accounts for survivorship bias than raw ICU LOS. When analyses were repeated excluding patients who died, the trend toward longer LOS in the CSA group persisted, though it remained nonsignificant. Taken together, our findings do not necessarily contradict prior studies but rather underscore the influence of survivorship and methodological differences in outcome reporting. Further research is warranted to clarify the impact of CSA on ICU resource utilization and recovery trajectories.

The CSA-treated group had on average 0.3 more procedures compared to non-CSA-treated patients, but this was not statistically significant. In the multiple linear regression, mortality reduced the number of procedures by 1.9. Similar to the LOS findings, the nonsignificant increase in number of procedures is likely explainable by the increase in mortality of the non-CSA-treated group despite controlling for mortality in the model. Existing studies have demonstrated a 60% reduction in number of grafting procedures compared to controls when CSA was used^14^ and economic analyses of adult data demonstrated cost savings, partly from reduced number of procedures, in the Burn Effectiveness Assessment Cost Outcomes Nexus (BEACON) model.^7^^,^^15^ The BEACON model used prior publications, surgeon interviews/surveys, and surviving patients from the National Burn Repository (NBR) database to inform the clinical inputs rather than actual discrete patient data as in the current study.

This study has several limitations and supports the need for further investigation. This study was limited by its retrospective nature and the data available in the de-identified burn registry at our institution. Missing sex, comorbidities, and other clinically relevant confounders likely influenced the results and conclusions. While the practice patterns at our institution during this period includes using CSA almost exclusively for widely meshed skin grafts rather than as primary autologous coverage for partial-thickness burns, it was not possible to definitively say that all patients included in the study received CSA for widely meshed grafts only. Due to these limitations, we attempted to minimize the influence of bias by controlling for third-degree TBSA and age through a 1:2 match, while also accounting for other variables in multiple linear and logistic regression. Despite these efforts, our results were likely influenced by selection bias of patients receiving CSA and chronology bias of patients from earlier years of the study period not receiving CSA or other more recent advances such as BTM. Nevertheless, the significant reduction in mortality and reduced odds of death observed in our CSA-treated group are suggestive of the positive impact that CSA treatment has on improving patient mortality. Ideally, a future study will be a prospective, randomized, controlled clinical trial, but a simpler and cost efficient design could be a retrospective multi-institutional study of actual electronic medical records to be able to analyze more granular data and to assess the impact of practice patterns which may vary institutionally.

## CONCLUSION

This study is one of the few studies that investigate the relationship between CSA treatment and patient with burn injury mortality, revealing a significant reduction in mortality and odds of death for patients treated with CSA compared to those not treated with CSA. Likely from this increased survivorship, CSA-treated patients also demonstrated an increase in LOS, ICU LOS, and number of procedures compared to controls, which is naturally expected with improvements in mortality. These promising results add to existing literature demonstrating how CSA can improve outcomes in patients with burn injuries. However, further research is necessary to validate these findings and to address the obvious biases inherent in this retrospective study.

## Notes

Author contributions: Muzamil Ahmad (Data curation, Investigation, Methodology, Writing—original draft, Writing—review & editing [lead]), Soman Sen (Writing—review & editing [supporting]), Kathleen Romanowski (Writing—review & editing [supporting]), Tina Palmieri (Writing—review & editing [supporting]), David G. Greenhalgh (Writing—review & editing [supporting]), and Jason Heard (Conceptualization, Data curation, Formal analysis, Investigation, Methodology, Project administration, Supervision, Writing—original draft, Writing—review & editing [lead])

Funding: None declared.
